# Ocular immune‐related diseases: molecular mechanisms and therapy

**DOI:** 10.1002/mco2.70021

**Published:** 2024-11-28

**Authors:** Yakun Wang, Shangze Gao, Fan Cao, Hui Yang, Fengyang Lei, Shengping Hou

**Affiliations:** ^1^ The First Affiliated Hospital of Chongqing Medical University Chongqing China; ^2^ Beijing Institute of Ophthalmology Beijing Tongren Eye Center Beijing Tongren Hospital, Beijing Ophthalmology & Visual Sciences Key Laboratory, Capital Medical University Beijing China

**Keywords:** age‐related macular degeneration, diabetic retinopathy, Graves’ ophthalmopathy, immune, uveitis

## Abstract

Ocular immune‐related diseases, represent a spectrum of conditions driven by immune system dysregulation, include but not limit to uveitis, diabetic retinopathy, age‐related macular degeneration, Graves’ ophthalmopathy, etc. The molecular and cellular mechanisms underlying these diseases are typically dysfunctioned immune responses targeting ocular tissues, resulting in inflammation and tissue damage. Recent advances have further elucidated the pivotal role of different immune responses in the development, progression, as well as management of various ocular immune diseases. However, there is currently a relative lack of connection between the cellular mechanisms and treatments of several immune‐related ocular diseases. In this review, we discuss recent findings related to the immunopathogenesis of above‐mentioned diseases. In particular, we summarize the different types of immune cells, inflammatory mediators, and associated signaling pathways that are involved in the pathophysiology of above‐mentioned ophthalmopathies. Furthermore, we also discuss the future directions of utilizing anti‐inflammatory regime in the management of these diseases. This will facilitate a better understanding of the pathogenesis of immune‐related ocular diseases and provide new insights for future treatment approaches.

## Graphical abstract

The molecular mechanisms underlying ocular immune‐related diseases is due to the dysregulation of immune responses. The unbalanced activation of diverse immune cell types, inflammatory activators and pathways are related to the immunological pathogenesis of ocular immune‐related disease which paly an essential role of immune processes in the development, progression and treatment of various ocular immune‐related diseases.

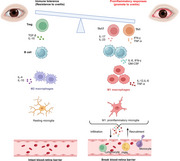

## 1 INTRODUCTION

Eyes are the primary mechanisms through which humans acquire external information, making ocular health a crucial component of overall well‐being. Immunological factors play a significant role in maintaining eye health; thus, a balanced ocular immune system is essential to maintain the homeostasis; however, any dysregulation may result in a myriad of ocular diseases. The eyes have long been regarded as “immune‐privileged” organs. The integrity of the blood–retinal barrier (BRB) provides an immune privilege to mammalian eye.^1^ This privilege is honored by a state of tolerance toward self‐antigens, which is further enhanced by the presence of endogenous immunosuppressive factors.^2^, ^3^, ^4^ Immunological mechanism in ocular tissues prevents or resolves inflammation and maintain homeostasis. Under different circumstances, skewed activation of the immune system induces autoimmunity, which affects the ocular and its surrounding tissues in certain extend.^1^

In general, ocular diseases span a wide range of pathologies with distinctive mechanisms. Recent progresses in the field of ocular immunology have preliminarily elucidated the complex interactions between immune components and ocular tissues. The discovery of immune checkpoint theory, the delineation of T and B‐cell function, and the exploration of genetic predispositions have further expanded our understanding of the pathophysiology of different ocular immune disease.^5^ Cellular mechanisms of immune response are playing an increasingly important role in the pathogenesis of immune‐related ocular diseases. In addition to T and B cells, the roles of microglia and other macrophages are also being intensively investigated.

In this review, we favored the discussion of uveitis, diabetic retinopathy (DR), age‐related macular degeneration (AMD), and Graves’ ophthalmopathy (GO) are due to the pivotal role that immune factors play in their pathogenesis. Interventions targeting these immune mechanisms have shown to yield positive therapeutic effects on these diseases. This approach is beneficial for broadening our comprehension of the intricate mechanisms underlying in ocular immune diseases significantly promotes the advancement for designing targeted therapies. The progression of targeted therapies is facilitated by using immunomodulatory agents and biologics, which have been sophisticatedly engineered to precisely modulate the immune response.^6^ Studies on the molecular mechanism of these immune responses could further open new avenues for disease treatment. Furthermore, investigations on the role of the BRB played in maintaining immune privilege also yields significant outputs.^7^ These findings are instrumental in blueprinting strategies that safeguard ocular integrity while effectively manage inflammations.

Immunotherapy using biologics and other targeting agents, which was stemmed from extensive understanding of disease pathogenesis, has become a novel strategy in treating immune‐related ocular diseases.^8^ Moreover, genetic studies have uncovered a group of susceptibility genes linked to ocular immune‐related pathologies, that could be used for early diagnosis and/or developing intervention approach. Due to the close association between ocular manifestation and systemic diseases, formulation of a comprehensive and multidisciplinary treatment of ocular immune‐related diseases is urgently required.

The research field of ocular immune‐related diseases is a rapidly evolving, with a deepening understanding of disease mechanisms, the development and testing of novel therapeutic approaches have been significantly expedite, aiming to improve treatment outcomes and patients’ quality of life.^9^

This review aims to provide a comprehensive overview of the current updates in ocular immunology, from the perspective of different immune cell types, this review provides a detailed exploration of the pathogenesis of ocular immune‐related diseases, including uveitis, DR, AMD, and GO. We summarize the shared features of specific immune cells across these diseases. Finally, we conclude with a summary of the therapeutic strategies employed in the treatment of these conditions.

## 2 UVEITIS

### 2.1 Definition and classification

**FIGURE 1:**
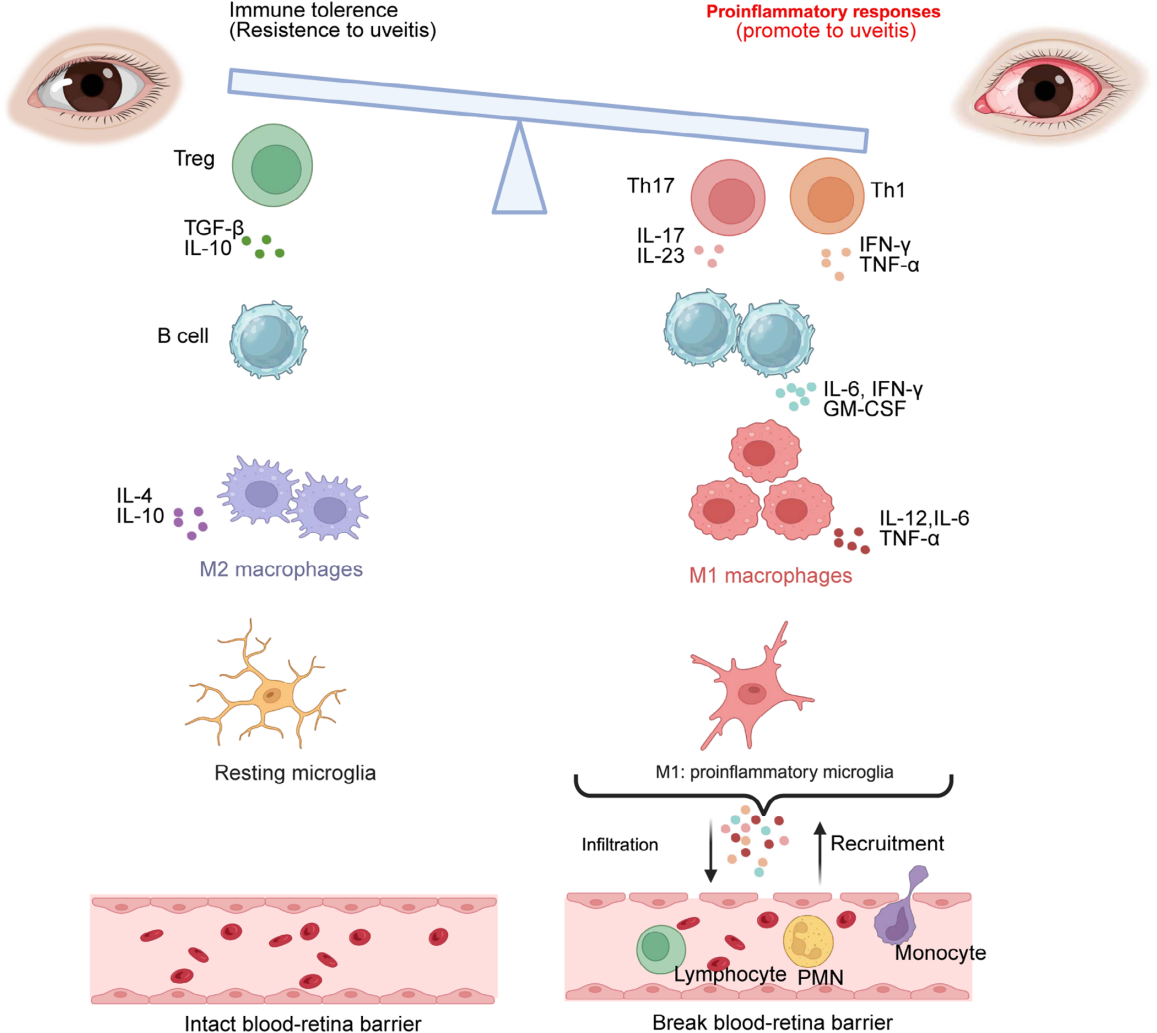
The dysregulation of immune response in uveitis. Uveitis results from imbalance between inflammatory mechanisms and regulatory mechanisms. Several immune cells are involved in this process. The balance between T helper 17 (Th17) cells and regulatory T cells (Tregs) is crucial for maintaining immune homeostasis and preventing the development of autoimmune disorders in uveitis. Effector B cells can produce pro‐inflammatory cytokines, such as interleukin‐6 (IL‐6), interferon‐γ (IFN‐γ), and granulocyte‐macrophage colony‐stimulating factor (GM‐CSF), which play a role in promotion of inflammation, and activation of other immune cells. M1 macrophages are typically associated with pro‐inflammatory responses and is crucial for the pathological of uveitis. The over activation of Th1/Th17, B cells and M1 macrophages results in the breaking down of the blood‒retinal barrier and, consequently, different inflammatory cells such as monocytes, granulocytes, and non‐specific lymphocytes from the circulation are recruited. These infiltrating inflammatory cells develop inflammation and further destroy the ocular tissue. IL‐4, interleukin‐4; IL‐10, interleukin‐10; IL‐12, interleukin‐12; IL‐17, interleukin‐17; IL‐23, interleukin‐23; PMN, polymorphonuclear; TGF‐β, transforming growth factor β; TNF‐α, tumor necrosis factor α.

Uveitis is a group of heterogeneous intraocular inflammatory diseases that extensively involve the uvea, retina, retinal vessels, and vitreous body.^10^ Uveitis is a leading cause of blindness worldwide and severely affects the vision of young people.^11^ It is reported that there are an estimated 3‒5 million patients with uveitis in China, and a 3%‒7% rate of blindness caused by it in Western developed countries.^12^, ^13^ Uveitis can be classified into infectious uveitis and non‐infectious uveitis (NIV). The latter is mainly related to the dysregulation of autoimmune or autoinflammatory responses (Figure 1), including autoimmune uveitis, idiopathic uveitis, uveitis associated with rheumatic diseases, and masquerade syndromes.^14^

### 2.2 Cellular mechanisms of immune response in uveitis

#### 2.2.1 T cell

T cells can be classified into several types based on their functions and surface markers: CD4^+^ T cells (helper T cells), which include T helper type 1 (Th1) cells, Th2 cells, and Th17 cells; CD8^+^ T cells (cytotoxic T cells); regulatory T cells (Tregs); memory T cells; and γδ T cells. Current research suggested that uveitis involved various immune cells, and the main pathogenic mechanism was due to the imbalance and dysfunction of T‐cell subsets.^15^ CD4^+^ T cells directed against self‐antigens play a significant role in the disease process, especially Th1 and Th17.^16^, ^17^ Tregs have a negative regulatory function on the immune system, can promote the resolution of inflammation, and are closely related to the outcome of uveitis.

Th1 cells are the main inflammatory cell groups in uveitis. Interferon‐gamma (IFN‐γ), as a characteristic cytokine of Th1 cells, is not expressed in the normal eye, but both IFN‐γ mRNA and protein can be detected in the inflammatory eyes of experimental autoimmune uveitis (EAU) and uveitis patients, and their expression is temporally related to the onset of uveitis, indicating that IFN‐γ is involved in the induction and pathogenesis of uveitis.^18^ Additionally, intraocular injection of IFN‐γ can lead to the recurrence of uveitis, suggesting that IFN‐γ may be involved in the recurrence of uveitis.^19^ The main inflammatory mediator of the Th1 cell response is tumor necrosis factor‐alpha (TNF‐α). The pro‐inflammatory cytokines interleukin‐2 (IL‐2) and IFN‐γ produced by Th1 cells are not directly toxic. During the inflammatory process, non‐specific white blood cells are activated to produce TNF‐α, which further promotes the activation of Th1 cells and is a key factor in causing inflammatory damage. In the EAU model, it has been found that levels of TNF‐α are significantly increased in the early stages of inflammation. Neutralizing TNF‐α can alleviate the inflammatory response, and treating in the early stages of inflammation can more effectively reduce the inflammatory response,^20^ indicating the role of TNF‐α in the initial stage of inflammation.

Th17 cells are a subset of helper T cells that secrete high levels of interleukin‐17 (IL‐17). Researchers have found that the expression of IL‐17 in inflamed eyes significantly increased after the induction of EAU. Adoptive transfer of autoreactive Th17 cells into healthy mice can induce uveitis symptoms, suggesting that Th17 cells can exert pro‐inflammatory effects independently of Th1 cells.^21^ The severity of EAU was significantly reduced in IL‐17 knockout or IL‐17 neutralizing treatment mice, highlighting its important role in disease progression. Our research established a new model of chronic relapsing EAU by adjusting peptide dosage and segments, and found that the response of memory Th17 cells predominates in the recurrence of EAU, indicating the important role of Th17 cells in the chronic inflammatory process of uveitis.^22^ Clinically, elevated levels of IL‐17 in the eyes and serum of patients with various NIV have also been observed,^23^ and the upregulation of IL‐17 was often associated with disease activity.^24^ Furthermore, interleukin‐23 (IL‐23) is closely related to Th17 cells. Mice with IL‐23 knockout had very low numbers of Th17 cells and were highly resistant to autoimmune and inflammatory responses, making it difficult to induce EAU.^25^, ^26^ Li et al.^27^ found that compared to young mice, the transcription and protein levels of the IL‐23 receptor in Th17 cells of aged mice were significantly reduced, and the inflammatory intensity of EAU induced in aged mice was lower. The serum levels of IL‐23 in Vogt‒Koyamagi‒Harada (VKH) patients and birdshot retinochoroidopathy patients were significantly elevated,^28^ suggesting that patients had a higher risk of developing uveitis.^29^ Genetically, our research^30^ also found that high gene copy numbers of IL‐17F and IL‐23A were related to the development of Behçet's disease and VKH syndrome. Researchers found that lactylation levels in CD4^+^ T cells increased in EAU and inhibition of lactylation reduced Th17 differentiation and attenuated inflammation in EAU.^31^ Lactylation in Th17 cells can influence the metabolism and epigenetic state of Th17 cells and alleviate inflammation by reducing the production of IL‐2 and IL‐17A.^32^

Tregs play a crucial role in maintaining self‐tolerance and regulating the body's immune response to infections and tumors. Tregs can negatively regulate the immune response to protect the body from chronic inflammatory damage. Tregs secrete transforming growth factor‐beta (TGF‐β) and interleukin‐10 (IL‐10), and their differentiation process requires the involvement of TGF‐β and specific transcription factors such as the Forkhead box P3 (FOXP3). It has been found that Tregs play a significant anti‐inflammatory role in EAU especially during the resolution phase where their numbers significantly increased, promoting the resolution of the inflammatory response.^33^, ^34^ IL‐10 and TGF‐β also exerted significant anti‐inflammatory activity in EAU. Treatment with IL‐10 after the induction of EAU could significantly reduce disease scores, while neutralization of IL‐10 could lead to an exacerbation of inflammation. TGF‐β was involved in the differentiation of Tregs considered a negative immune regulatory factor in EAU. In patients with uveitis and Behçet's disease, both the number of Tregs and the levels of TGF‐β were significantly reduced.^24^, ^35^, ^36^ Therefore, Tregs could promote disease remission and inhibit autoimmune reactions, providing new insights for the exploration of the mechanism of recurrent uveitis and clinical diagnosis and treatment.

The balance between Th17 cells and Tregs is crucial for maintaining immune homeostasis and preventing the development of autoimmune disorders. Th17 cells/Tregs ratio was significantly correlated with disease scores in human autoimmune uveitis and the EAU model. The restoration of the Th17 cell/Tregs ratio was often associated with the resolution of inflammation.^37^, ^38^ Therefore, inducing the conversion of Th17 cells into Tregs to reset the Th17 cell/Treg balance may be a promising therapeutic strategy in uveitis.

#### 2.2.2 B cells

NIU is believed to be autoimmune or mediated by immune factors.^39^ B cells possess a variety of potent immunological functions, such as secreting antibodies and cytokines, and antigen presentation.^40^ While the role of B cells in uveitis is not as well‐defined as those of T cells, increasing evidence indicates that B cells are not just bystanders; they play a significant role in NIU. Immunohistochemical findings show that B cells and plasma cells infiltrate in EAU and gradually become dominant in the later stages.^41^ This could be related to the breakdown of the blood‒ocular barrier in the advanced stages of the disease.

B cells can differentiate into antibody‐producing plasma cells, which bind to antigens on target cells to form immune complexes and exert biological effects. Due to the large molecular weight of antibodies, they have difficulty crossing the blood‒ocular barrier to enter the eye; hence, B cells only play a significant pathogenic role after the barrier is compromised in the late stages. Using bioluminescence technology to monitor the dynamics of immune cells in the eye, it has been found that B cells only increase significantly after 28 days of EAU induction.^42^ The increase in the number of B cells infiltrating the eye may be related to the prolongation of disease duration and exacerbation of the condition. Interestingly, administering B‐cell depleting agents 7 days before and after EAU induction yields completely different results, indicating that B cells have a protective role in the early stages and then shift to a pathogenic role.^43^ This further illustrates the complexity of B cells in the pathogenesis of NIU.

Effector B cells can produce pro‐inflammatory cytokines, such as interleukin‐6 (IL‐6), IFN‐γ, and granulocyte‐macrophage colony‐stimulating factor. The production of these cytokines by effector B cells could significantly contribute to the pathogenesis of autoimmune and inflammatory diseases. After the IL‐6 gene was knocked out in EAU mice, the ocular inflammatory response was significantly reduced.^44^ IL‐6 can regulate the balance of Treg/Th17,^45^ and can also induce the differentiation of follicular helper T cells (Tfh).^46^ B cells, in conjunction with T cells, monocytes, dendritic cells, and others, secrete large amounts of IL‐6, creating an inflammatory microenvironment, amplifying the immune response in a cascade manner, and ultimately contributing to the occurrence of the disease.

In patients with NIU, B cells and/or plasma cells were found to infiltrate the choroid, iris, and ciliary body. Although most cases were predominantly characterized by T cells, in certain situations, B cells could take a dominant role.^47^, ^48^ In the serum of NIU patients, increased levels of inflammatory cytokines, such as TNF‐α, IL‐6, and B‐cell‐related factors (including BAFF, APRIL, BCMA, and BlyS) were observed.^49^, ^50^, ^51^ These elevated levels of cytokines and B‐cell‐activating factors suggest a heightened inflammatory state and the involvement of B cells in the immune response associated with NIU.

#### 2.2.3 Microglia

Microglia are tissue‐specific macrophages that are localized in the central nervous system (CNS) and the retina. As resident immune cells, microglia play a crucial role in uveitis.^52^ Microglia were capable of quickly responding to changes in the retinal environment and maintaining homeostasis through various post‐translational modifications.^53^, ^54^, ^55^ In addition, there were reports that under inflammatory conditions, microglia could improve their chemotaxis through phosphorylation‐mediated protein activity regulation, while also enhancing the release of inflammatory cytokine.^56^, ^57^ In the past years, our team has been dedicated to studying the role of microglia in the pathogenesis of uveitis and the development of treatment methods. We found microglia constituting the largest proportion in retinal immune cells in EAU mice using single‐cell RNA sequencing.^58^ Our study showed that microglia exposed to hypoxic conditions rapidly accumulate lactate, which promoted lactylation of transcription factor YY1 at the lysine 183 (K183) site, enhancing angiogenesis in uveitis.^59^ Moreover, YY1 lactylation aggravated autoimmune uveitis by enhancing microglial functions via inflammatory genes and targeting the lactate/p300/YY1 lactylation/inflammatory genes axis may serve as a promising therapeutic strategy.^60^ Furthermore, modulation on the retinal microglial phenotype by transcription factor EGR2 through activation of GDF15 alleviated autoimmune uveitis.^61^

The suppression of Galectin‐3 reduced microglial reactivity and inflammatory reactions via the TLR4/MyD88/NF‐κB signaling cascade in autoimmune uveitis.^62^ Aryl hydrocarbon receptor (AhR) activation exhibited an immunomodulatory effect in EAU through modulation of macrophages/microglia polarization and the downregulation of nuclear factor kappa B (NF‐κB) and signal transducers and activators of transcription (STAT) pathways.^63^ The gene product of fat mass and obesity‐associated (FTO) mitigated the severity of autoimmune uveitis by modulating microglial phenotypes through the GPC4/TLR4/NF‐κB signaling pathway.^64^ In our in vivo studies, both icariin and apigenin were observed to markedly decrease clinical and pathological manifestations of experimental EAU through the suppression of microglial M1 polarization mediated by the TLR4/MyD88 signaling pathway.^65^, ^66^ Summarily, we found that microglia are the initiators and determinants of uveitis, clarified the regulatory network and mechanism of action of lactate and lactate modification in mediating microglial immune response, and proposed a new theory of microglia driving uveitis. Additionally, the functionality of microglia in the context of autoimmune uveitis is not only directly influenced by immunological and pathological conditions but also significantly shaped by genetic and environmental factors. Recent advances in genomics have highlighted the role of genetic factors in modulating microglial functions.^67^, ^68^ Therefore, all these studies proved that regulation of microglia will be another option to relieve the over‐inflammatory response in uveitis.

#### 2.2.4 Macrophages

Uveitis primarily occurs due to an imbalance in the regulation between the immune system and inflammatory mechanisms.^69^ The inflammation mediated by immune dysregulation is related to the activation of macrophages. They can participate in the development of uveitis through various pathways. Macrophages defend against the development of uveitis by activating into cells with different phenotypes.^70^

Macrophage activation is a process that can respond to stimulus signals in the immune system microenvironment with certain immune regulatory reactions. Macrophages exist in two activation states: classically activated macrophages (M1) and alternatively activated macrophages (M2).^71^ M1 macrophages are typically associated with pro‐inflammatory responses and are involved in the clearance of pathogens and the initiation of adaptive immunity by presenting antigens to T cells. They produce high levels of pro‐inflammatory cytokines such as IL‐12, IL‐6, and TNF‐α, which can promote Th1 responses.^72^ M2 macrophages are associated with anti‐inflammatory and tissue repair processes and response to stimulation by IL‐4 and IL‐10.^73^ Different stimuli induce different subtypes of M2, which can reduce T‐cell antigen presentation and promote the production of cytokines that stimulate Th2 responses. As a result, the immunosuppressive phenotype is more pronounced than in their baseline state. The balance between M1 and M2 activation states is crucial for the proper functioning of the immune response and the prevention of pathological conditions, including uveitis.

Plasticity of macrophages allows them to be both pro‐inflammatory and anti‐inflammatory. Studies have identified a significant association between uveitis and specific chemokines, including neutrophil chemotactic factor (CXCL8), monocyte chemoattractant protein 1, and macrophage inflammatory protein 1β, which are implicated in the disease's etiology and progression. Under specific conditions, CXCL8 could regulate the endothelial adhesion, chemotaxis, and activation of other leukocytes,^74^ affecting the secretion of inflammatory factors by monocytes and T lymphocytes, thereby causing local infiltration of macrophages and leading to uveitis.

Recently, animal experimental studies on uveitis have indicated that regulating macrophage polarization is of great significance for its targeted diagnosis and treatment. Wang et al.^75^ used Longdan Xiegan Tang (a traditional Chinese medicine) to reduce the polarization level of M1‐type macrophages and the expression level of inducible nitric oxide synthase (iNOS) while increasing the polarization level of M2‐type macrophages and the expression level of arginase‐1 (Arg‐1). In the study by Qu et al.^76^ miR‐223‐3p can negatively regulate the Nod‐like receptor family pyrin domain‐containing protein 3 (NLRP3), affecting the progression of uveitis. By increasing the expression level of miR‐223‐3p, the anti‐inflammatory effect of M2 macrophages is enhanced, thereby reducing the inflammatory response in uveitis. This research highlights the potential of modulating macrophage polarization as a therapeutic strategy in uveitis. Currently, a large number of experiments both domestically and internationally have shown that the NLRP3 inflammasome plays an important regulatory role in the pathogenesis of uveitis.^77^, ^78^ Our research confirmed that macrophages show an increased M1 activation and pyroptosis in Nlrp3^‒/‒^ mice which was mediated by the upregulated transcription of Aim2 because of Nlrp3 deficiency.^79^

By shifting the balance between M1 and M2 macrophages, it may be possible to control the inflammatory process and promote the resolution of inflammation, which could be beneficial in the management of uveitis.

### 2.3 Treatment

Management of NIV typically involves local treatment strategies, such as topical corticosteroids, or regional administration via injections or implants. Systemic interventions may also be employed, encompassing oral corticosteroids, immunosuppressants, and biological therapies. The choice of therapy is often guided by the primary site of inflammation within the eye.

Corticosteroids are currently the traditional first‐line therapy for NIU, with administration routes including topical drops, intravitreal injections, systemic oral, or intravenous (IV) injections.^80^ They function by curbing the production of inflammatory mediators and enhancing the levels of anti‐inflammatory counterparts, thereby mitigating inflammation. Additionally, they help to quell delayed‐type hypersensitivity responses. Nonetheless, their prolonged high‐dose administration is constrained by the potential for adverse effects.

Immunosuppressants, in cases of uveitis that are difficult to control with steroids, physicians may recommend the use of immunosuppressants to suppress the inflammatory response. Commonly used immunosuppressants include azathioprine, cyclosporine, cyclophosphamide, and methotrexate.^81^, ^82^

The advent of biologic medications has provided new therapeutic avenues for treating NIU. Adalimumab and infliximab which is TNF‐α inhibitors, are the most used biologic agents in these patients. After more than 20 years of exploration, biological inhibitors have gradually become a new option for patients with refractory NIU or those intolerant to conventional treatments.^83^ TNF antagonists have a specific role in inflammatory pathogenesis and are currently widely used as biological agents. Some authorities advocate for the simultaneous administration of a traditional immunosuppressive medication, such as methotrexate or mycophenolate mofetil, alongside infliximab to diminish the likelihood of developing anti‐infliximab antibodies. Moreover, an increasing number of clinical trials have been conducted recently to study the immune therapy for NIU (Table 1). The treatment method for autoimmune eye diseases still needs further exploration.

**TABLE 1:** List of clinical trials for uveitis immune therapy in decade.

Study ID	Patients	Drugs	Phases	Year	Country
NCT03886233	Autoimmune uveitis	Corticosteroid series	NA	2024	China
NCT06310837	Uveitis	Adalimumab, corticosteroid	NA	2024	China
NCT00132691	Uveitis	Corticosteroid, immunosuppressive agents	IV	2024	USA
NCT02252328	Uveitis	Simvastatin	II	2024	UK
NCT02049476	Uveitis	Dexamethasone pellet	IV	2024	USA
NCT00646425	Non‐infectious uveitis	Basiliximab	II	2024	USA
NCT01232920	Uveitis	Methotrexate, mycophenolate mofetil	III	2024	India
NCT03828019	Uveitis	Adalimumab	III	2019	USA
NCT01900431	Uveitis	Sarilumab, prednisone, methotrexate	II	2024	USA
NCT03097315	Uveitis	CLS‐TA suprachoroidal injection	III	2017	USA
NCT00043667	Uveitis	Daclizumab	II	2024	USA
NCT03399175	Vogt‒Koyanagi‒Harada	Corticosteroid, immunosuppressive therapy	NA	2015	Brazil
NCT01789320	Uveitis	Triamcinolone acetonide	I	2024	USA
NCT01280669	Uveitis	Sirolimus	II	2024	USA
NCT01279954	Uveitis	Abatacept	II	2024	USA
NCT00803816	Uveitis	Everolimus	II	2024	Germany
NCT00908466	Uveitis	Sirolimus	I	2024	USA
NCT04798755	Uveitis	Methotrexate, adalimumab	III	2022	Spain
NCT04588818	Uveitis	Adalimumab, methotrexate	II	2020	China
NCT00876434	Anterior uveitis	Sirolimus	I	2009	USA
NCT03209219	Behçet disease, uveitis	Interferon‐α‐2A, cyclosporine	III	2017	China
NCT05015335	Uveitis	Adalimumab, methotrexate	IV	2021	China
NCT02623426	Uveitis, macular edema	Dexamethasone, methotrexate, ranibizumab	III	2017	USA
NCT00012506	Uveitis, arthritis	TNFR: Fc	III	1905	USA
NCT00130637	Anterior uveitis	Daclizumab	II	2024	USA
NCT01314417	Non‐infectious uveitis	Methotrexate	I	2024	USA
NCT01791192	Non‐infectious uveitis	Oral corticosteroid	II	2024	USA
NCT00344253	Uveitis, multiple sclerosis	Interferon‐β, methotrexate	III	2024	Germany
NCT00918554	Uveitis, ocular sarcoidosis	Methotrexate	IV	2024	France

Abbreviations: TA, triamcinolone acetonide; TNFR: Fc, tumor necrosis factor receptor‐Fc.

*Source*: www.clinicaltrials.gov.

## 3 DIABETIC RETINOPATHY

### 3.1 Definition and classification

**FIGURE 2:**
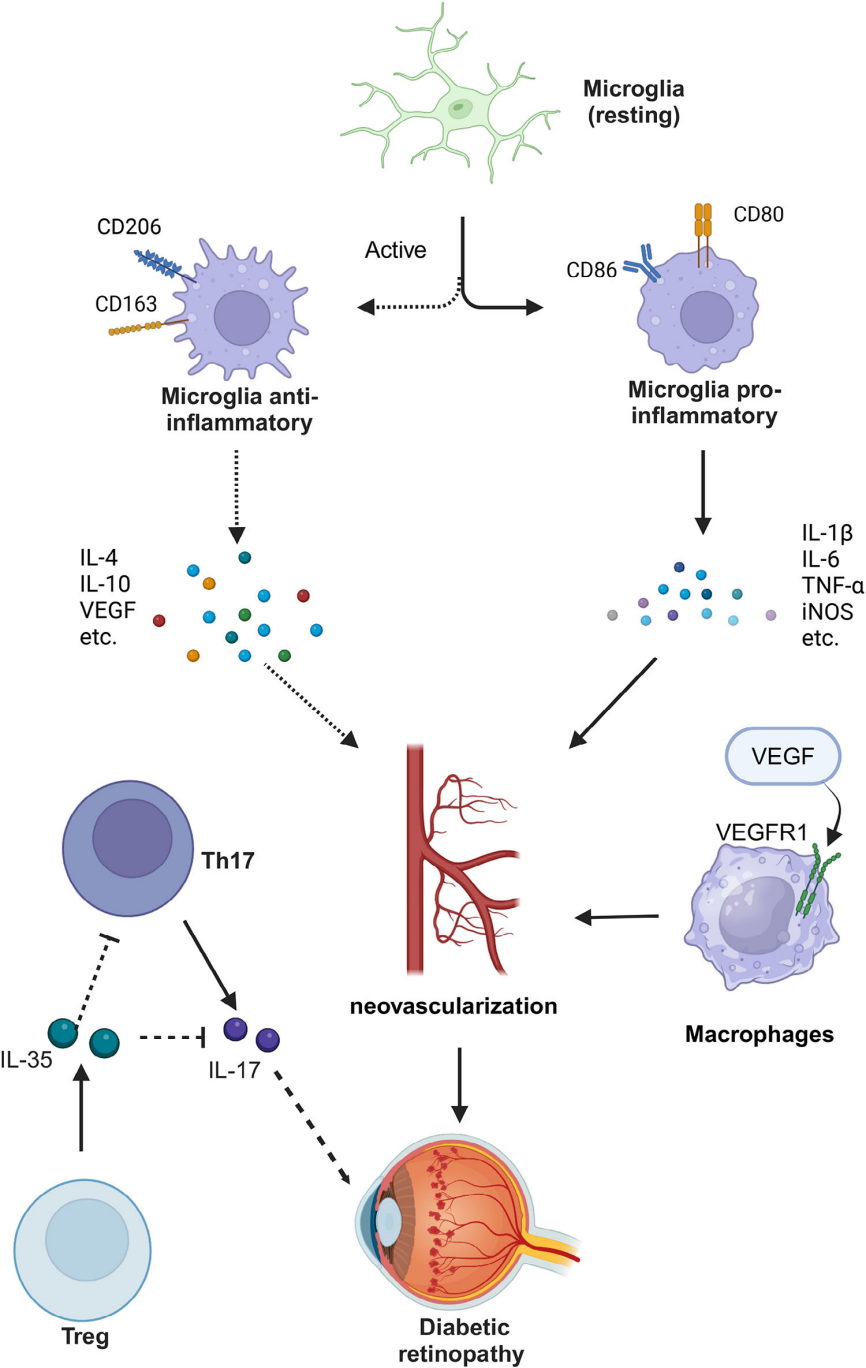
The role of immune cells in the pathogenesis of diabetic retinopathy. Microglial polarization into M1 and M2 phenotypes releases a multitude of inflammatory factors, and the action of vascular endothelial growth factor (VEGF) on vascular endothelial growth factor receptor 1 (VEGFR1) in macrophages can induce neovascularization, leading to diabetic retinopathy (DR). Furthermore, the interaction between T helper 17 (Th17) cells and regulatory T cells (Tregs) may also exacerbate the progression of DR. IL‐35, interleukin‐35; iNOS, inducible nitric oxide synthase.

DR is one of the most common complications of diabetes and is a leading cause of visual impairment and blindness worldwide. DR affects populations of all ages globally, with a prevalence of 34.6% (93 million) in adults aged 40 and above.^84^ DR can be classified into non‐proliferative diabetic retinopathy (NPDR) and proliferative diabetic retinopathy (PDR) based on its stages of development and severity. PDR is more severe and typically involves the formation of new blood vessels, vitreous hemorrhage, and one or more types of bleeding in the front of the retina.^85^ The onset and escalation of DR are characterized by an intricate network of pathogenic and physiological factors, encompassing oxidative stress triggered by hyperglycemia, immune system activation, inflammation, impairment of vascular endothelium, and retinal neuronal injury.^86^, ^87^, ^88^ Increasing evidence suggests that immune factors play an increasingly important role in the pathogenesis of DR (Figure 2).

### 3.2 Cellular mechanisms of immune response in DR

#### 3.2.1 T cell

The role of T cells in DR is receiving increasing attention. Th17 cells have been reported to play a significant role in the pathogenesis of DR. Th17 cells belong to the CD4^+^ T‐cell lineage and are known to regulate the secretion of IL‐17A, which is closely associated with the occurrence of inflammation and autoimmune diseases.^89^ Previous reports have demonstrated that elevated levels of IL‐17 produced by Th17 cells were associated with DR. In gene knockout mice, increased secretion of IL‐17 resulted in significantly exacerbated lesions of DR.^90^ Takeuchi et al.^91^ found that the levels of IL‐17 in the vitreous fluid of patients with PDR were significantly higher than those in non‐DR patients. These findings provide strong evidence that increased IL‐17 levels are correlated with the severity of DR.

In addition to the effects of Th17 cells themselves, the interaction between Th17 cells and Tregs also plays a significant role in the progression of DR. IL‐35, secreted by Tregs, acts as an immunosuppressive factor in various inflammatory responses.^92^ Studies have reported that the levels of IL‐35 in the serum and vitreous of patients with PDR were reduced.^93^ Furthermore, IL‐35 has been shown to inhibit the levels of IL‐17 and reduce the infiltration of CD4^+^ T cells, thereby preventing inflammatory bowel disease and psoriasis.^94^ In the context of DR, IL‐35 can also lower IL‐17 levels and suppress Th17 differentiation, exerting a protective effect against PDR.^95^ This clearly illustrates the significant interplay between Th17 cells and Tregs in the pathogenesis of DR.

#### 3.2.2 Microglia

Microglia, being the primary immune cell population resident in the CNS, are pivotal in the modulation of retinal angiogenesis under both physiological and pathological contexts of DR. In a healthy state, a subset of microglia remains in a resting condition, characterized by the purinergic receptor P2RY12, which is involved in their surveillance function and is considered a specific marker for resting microglia. The activation of microglia was classified into two phenotypes, M1 and M2. M1 microglia were considered pro‐inflammatory, while M2 microglia were regarded as anti‐inflammatory or reparative cells. M1 microglia are distinguished by their expression of markers such as iNOS, CD16, and CD32, as well as their secretion of pro‐inflammatory cytokines such as IL‐1β, IL‐6, and TNF‐α. In contrast, M2 microglia were identified by markers such as CD206, Arg1, and Ym1, and they release anti‐inflammatory cytokines such as IL‐4 and IL‐10.^96,^ ^97^ In DR, microglia are extensively activated, exhibiting a pronounced polarization phenotype, as the progression of DR occurs, microglia transition from the M2 phenotype to the M1 phenotype.^98^ In pathological states, not only does the polarization of microglia occur, but the number and distribution of polarized microglia also significantly differ from those under physiological conditions. Microglia in the neovascularization tuft region predominantly display an activated state. Researchers have found that in the neovascularization tuft region, more than 80% of microglia were activated, whereas under normal conditions, the proportion of activated microglia was less than 2%.^99^ Under pathological conditions, not only are microglia polarized in vivo, but in vitro studies have shown that the activation of microglia in hypoxic and inflammatory environments can lead to their transformation into an angiogenic phenotype. In in vitro models under LPS exposure and hypoxic culture conditions, the secretion of pro‐angiogenic and inflammatory factors by microglia was significantly increased.^100^, ^101^

In the pathogenesis of DR, the role of inflammation has been increasingly recognized as pivotal. It is well‐established that microglia are activated under pathological conditions to secrete a plethora of inflammatory cytokines. Moreover, elevated levels of IL‐1, IL‐6, IFN‐γ, and TNF‐α can also activate microglia, creating a feedback loop that facilitates the rapid and extensive activation of these cells.^102^, ^103^ A substantial body of research has demonstrated elevated levels of inflammatory cytokines in the bodily fluids of patients with DR. For instance, Zorena et al.^104^ reported that serum levels of TNF‐α, IL‐6, and C‐reactive protein were significantly higher in children with type 1 diabetes mellitus (DM) and NPDR compared to those without retinal pathology. Feng et al.^105^ have observed that in the aqueous humor of patients with DR, the levels of inflammatory cytokines, including IL‐1β, IL‐6, IL‐8, IL‐17A, and TNF‐α, were significantly elevated compared to patients without DR. Wu et al.^106^ have reported a significant correlation between the levels of IL‐8, placental growth factor (PlGF), and vascular endothelial growth factor (VEGF) in both the vitreous and aqueous humor of patients with PDR. Doganay et al.^107^ have observed that the mean serum levels of NO, soluble interleukin‐2 receptor (sIL‐2R), IL‐8, and TNF‐α increased with the progression of DR, with the highest levels being detected in patients with PDR. Csősz et al.^108^ have reported that in tear samples, the concentrations of lipocalin‐1, lactotransferrin, lacritin, lysozyme C, lipophilin A, and immunoglobulin lambda chain were significantly elevated in patients with DR.

In addition to the aforementioned inflammatory cytokines and protein markers, VEGF is one of the most well‐established biomarkers associated with the angiogenic processes in DR.^109^ Five VEGFR ligands (VEGF‐A, ‐B, ‐C, ‐D, and PlGF) constitute the VEGF family.^110^ The most characteristic process involved the activation of VEGFR2 tyrosine kinase (TK) by VEGF‐A in endothelial cells, thereby inducing angiogenesis and increasing vascular permeability.^111^ In microglia, studies have identified that VEGF‐B produced by these cells can exacerbate the progression of autoimmune diseases.^112^ Under pathological conditions, microglia express VEGFR1 and subsequently upregulate pro‐inflammatory and pro‐angiogenic cytokines, such as VEGF‐A and PlGF, thereby promoting retinal angiogenesis.^113^ Consequently, in the context of DR, the polarization of microglia leads to an increase in the expression of VEGF. In turn, elevated levels of VEGF further enhance the activation of microglia, creating a positive feedback loop that ultimately results in the formation of retinal neovascularization and exacerbates the progression of DR.^114^, ^115^, ^116^ In summary, microglia play a pivotal role in the pathogenesis and progression of DR by modulating a range of protein factors and inflammatory mediators.

#### 3.2.3 Macrophages

Ocular macrophages include both resident populations, such as retinal microglia, perivascular macrophages, and hyalocytes on the retinal surface, as well as macrophages originating from infiltrating monocytes.^117^ In the preceding section, we have thoroughly elucidated the role and mechanisms of microglia in DR. In the following sections, we will analyze several other types of macrophages. It is well known that macrophages play a crucial role in the process of pathological neovascularization. Ogura et al.^113^ found that macrophage‐derived VEGF and PlGF activated VEGFR1 in macrophages and VEGFR2 in endothelial cells. Shen et al.^118^ discovered that succinate was a novel angiogenic factor that can regulate the M2 polarization of macrophages and modulate the RBP4/VEGFR2 pathway, thereby inducing pathological angiogenesis. Yamaguchi et al. found that during DR, the increased expression of VEGF promoted the activation of vitreous resident macrophages, which in turn recognized intraretinal hyperreflective foci associated with DR.^119^ These studies provided evidence that other types of macrophages can also produce or act through factors of the VEGF family, leading to neovascularization. The transparent cells on the retinal surface also play a crucial role. These cells, known as vitreous‐resident macrophages, demonstrate responsiveness to antigens, additionally, they maintain the transparency of the vitreous by clearing cellular debris and secrete anti‐angiogenic factors to inhibit vascular growth under stable conditions.^120^, ^121^ Mendes‐Jorge et al.^122^ have discovered the presence of auto fluorescent macrophages surrounding the retinal vasculature. These cells, distinct from microglia, possess scavenger functions and may contribute to the maintenance of the BRB in a healthy state, as well as being implicated in the pathogenesis of retinal diseases. Similarly, Mato et al.^123^ identified specific macrophages surrounding arterial blood vessels that were involved in barrier functions and phagocytic activities in the cerebral cortex. To date, the role of perivascular macrophages in DR has been minimally investigated, and the specific mechanisms of action remain to be further explored.^124^

### 3.3 Treatment

The therapeutic modalities for DR are diverse. For instance, pan retinal photocoagulation (PRP) is a commonly employed surgical intervention that treats DR by utilizing high‐energy light to destroy neovascularization.^125^ PRP may be associated with permanent scarring and vision loss due to damage to healthy retinal tissue.^126^ Anti‐VEGF agents are gaining popularity as non‐surgical treatment options, which can be used alone or in conjunction with PRP.^127^ However, concerning the current treatment efficacy, anti‐VEGF therapy has been shown to have limited effectiveness and potential side effects. Moreover, studies have reported that in the DR population, a subset of cases cannot be effectively resolved even with repeated injections of anti‐VEGF agents.^128^ This could be due to the overlooked or suboptimal management of inflammatory elements. Administration of intraocular steroids is an alternative therapeutic strategy, especially for individuals with insufficient responses to anti‐VEGF treatments.^129^ However, the associated more severe side effects of steroid medications, such as an increased risk of glaucoma and cataracts, underscore the urgent need to develop a novel and more optimal treatment approach.

Targeting microglial cells with pharmacological agents has been identified as a potential therapeutic strategy for DR.^130^ Research suggested that targeting microglia and their polarization states may represent a promising adjunctive approach for treating DR, offering new alternative pathways beyond traditional treatment methods.^131^, ^132^, ^133^ Xu et al.^134^ have discovered that in the animal model of neovascularization, melatonin was capable of reverting the activation of a subset of microglia to a quiescent state, which consequently mitigated the formation of retinal neovascularization. Wang et al.^135^ found that the selective TAK‐1 inhibitor 5Z‐7‐oxozeaenol significantly reduced the number of activated microglia and attenuated abnormal retinal angiogenesis in the OIR model through the inhibition of TAK‐1. Cyanidin‐3‐O‐glucoside (C3G), HIF‐1α inhibitor (KC7F2), and Magnolol have been shown to exert inhibitory effects on retinal neovascularization by reducing the activation of microglia.^136^, ^137^, ^138^

In addition to inhibiting microglial activation, promoting the polarization of microglia from the M1 to the M2 phenotype is also a therapeutic strategy for DR. However, there are reports indicating that an excessive proportion of M2‐polarized microglia may promote pathological retinal neovascularization rather than exerting an anti‐angiogenic effect.^139^ Therefore, maintaining a balanced ratio of mixed M1 and M2 microglia is crucial for inhibiting neovascular formation. Sun et al.^140^ discovered that ferulic acid alleviated inflammatory responses and achieved anti‐angiogenic effects by modulating the polarization of microglia from M1 to M2. It is well known that microglial cells can secrete retinal angiogenic factors such as VEGF‐A, TNF‐α, and IL‐1β, leading to the formation of neovascularization during the progression of DR. Therefore, measures aimed at blocking microglial secretion of these angiogenic factors are likely to have a therapeutic effect on DR. Compounds such as chlorogenic acid and erianin have been shown to reduce VEGF‐A expression in microglia by inhibiting the transcriptional activation of HIF‐1α, effectively decreasing retinal angiogenesis and alleviating the progression of DR.^141^, ^142^ Omega‐3 polyunsaturated fatty acids (ω‐3‐PUFA) can inhibit retinal neovascularization by reducing TNF‐α production from microglia and suppressing microglial pyroptosis.^143^, ^144^

In addition to interventions targeting macrophages, modulation of other cell types can also yield therapeutic effects. Berberine has been shown to alleviate DR by adjusting the Th17/Tregs ratio, specifically by reducing Th17 cell populations and increasing Treg cells.^145^ The aryl hydrocarbon receptor agonist VAF347 may exert therapeutic effects on DR by regulating the secretion of Th17 cells and IL‐17.^146^

## 4 AGE‐RELATED MACULAR DEGENERATION

### 4.1 Definition and classification

AMD is the most common cause of severe loss of eyesight among people over 60 years,^147^ and the number of global AMD patients is predicted to reach more than 3 million cases by 2040.^148^ AMD was classified as two types of macular degeneration by the Classification Committee of the Beckman Initiative for Macular Research: dry (non‐exudative or atrophic) and wet (exudative or neovascular). All AMD begins in the dry form and approximately 85% of AMD patients have only the dry form of AMD. Patients with wet AMD account for about 15%. Although only 15% of AMD patients have the wet form, 80%‒90% of severe vision loss is caused by wet AMD.^149^ Numerous studies have linked aging, obesity, smoking, hypertension, cardiovascular disease, and genetic factors to more severe AMD.^150^, ^151^, ^152^

### 4.2 Molecular mechanisms of immune response in AMD

The pathogenesis of AMD is complex, involving cellular senescence and immune homeostasis dysregulation.^153^ Recent advances have highlighted the essential role of immune processes in the development, progression, and treatment of AMD.^154^, ^155^, ^156^ While a properly functioning retinal immune system is essential for maintaining visual homeostasis, an abundance of evidence suggests that excessive activation of certain immune responses significantly contributes to the development of AMD.^156^

#### 4.2.1 T cell

T lymphocytes, integral to the adaptive immune system, have a significant role in AMD. Helper T (Th) cells are particularly influential; they can activate B cells for antibody production, macrophages for pathogen destruction, and cytotoxic T cells to eliminate infected cells.^157^ In AMD, T cells secrete pro‐inflammatory cytokines, such as those found in the carboxyethyl pyrrole‐specific T‐cell response, which contributes to M1 macrophage polarization. This interaction is a critical early link between innate and adaptive immunity in AMD pathogenesis. An increase in Th1 cytokines in the vitreous and aqueous humor was observed in AMD, and a lower frequency of Th1 cells and CXCR3^+^CD4^+^ T cells in neovascular AMD (nAMD) patients suggested a potential contribution to angiogenesis and CNV.^158^, ^159^ Th2 and Th17 cells are thought to play a role in subretinal fibrosis development. Elevated levels of Tfh cells in AMD patients enhanced B‐cell antibody production, while an increase in circulating senescent CD56^+^CD28^‒^ T cells in nAMD indicated a link to T‐cell immunosenescence. The interplay between T cells, cytokines, and macrophages is intricate and influential in the progression of AMD. Understanding these dynamics is crucial for developing targeted therapies that address the adaptive immune response in AMD.

#### 4.2.2 Macrophages

Microglia is the resident immune cells of the retina and preserve normal retinal function. Their duties encompass surveillance and the phagocytosis of cellular debris from damaged cells, which are essential for retinal homeostasis.^160^ However, with aging, microglial responsiveness to injury diminishes, and the ensuing dysfunction of these cells is increasingly recognized as a pivotal element in the early stages of AMD.^161^, ^162^, ^163^ Microglia's subretinal migration is essential for photoreceptor cell survival, yet impaired migration can lead to cell death.^164^ In the wet form of AMD, microglia and macrophages contribute to neovascular lesion growth by releasing pro‐angiogenic factors such as VEGF‐A.^163^, ^165^ These cells, normally absent in the retina, reside in the choroid and are recruited to the retina during BRB breakdown, modulating disease severity.^166^

In the dry form of AMD, macrophages are involved in the clearance of metabolic waste between retinal pigment epithelial (RPE) cells, especially the lipid and protein complexes that accumulate on Bruch's membrane, known as drusen. The accumulation of these deposits is associated with the dysfunctional clearance by macrophages, which can lead to an inflammatory response damaging RPE cells and photoreceptors, resulting in vision decline.^167^ The role of macrophages in wet AMD is more complex. They contribute to the formation of pathological neovascularization and may promote disease progression by releasing angiogenic factors. Additionally, macrophages are implicated in the inflammation and leakage of abnormal blood vessels, potentially causing retinal edema, hemorrhage, and scarring, leading to severe vision loss.^168^

The polarization of macrophages into M1 (pro‐inflammatory) and M2 (anti‐inflammatory) phenotypes significantly influences AMD's pathology. M1 macrophages are linked to initial choroidal neovascularization (CNV) stages, while the M2 macrophages are crucial in CNV development and remodeling. Yang et al.^169^ have shown that M1 macrophages were involved in the early phase of CNV, whereas the M2 phenotype exerted a significant influence on the intermediate and later stages of CNV evolution and remodeling. Consequently, the M2 phenotype was deemed more critical in the advancement of CNV. However, Zhou et al.^170^ have reported that M1 macrophages exerted a more pronounced inhibitory effect on CNV progression, with M1 macrophages predominantly residing in the RPE‐choroid and M2 macrophages primarily situated in the retina. The recruitment of macrophages to the Bruch'membrane(BrM) and the polarization of resident choroidal macrophages were associated with extracellular deposits, such as soft drusen and extensive basal laminar deposits.^171^ The ROCK signaling pathway's influence on macrophage polarization suggests a connection between aging and the overexpression of pro‐angiogenic M2 macrophages.^172^

#### 4.2.3 Neutrophils

Neutrophils play a vital role in AMD, particularly wet AMD, where elevated neutrophil‐to‐lymphocyte ratios (NLR) are linked to disease severity. They contribute to retinal angiogenesis by producing MMP‐9, which degrades the extracellular matrix, a critical step in the process.^173^, ^174^ Additionally, they release pro‐angiogenic factors VEGF and IL‐8, amplifying their recruitment and Matrix metalloproteinase‐9 (MMP‐9)secretion. The presence of lipocalin‐2 (LCN‐2)‐positive neutrophils in early AMD suggests a role in disease initiation, with the AKT2/NF‐κB/LCN‐2 signaling axis implicated in inflammatory activation. Targeting AKT2 has shown promise in reducing LCN‐2‐driven neutrophil infiltration and reversing early AMD phenotypes.^175^

#### 4.2.4 Complement system

The complement system, with its extensive network of proteins, is central to innate immunity and has a dual role in AMD, as supported by genetic studies.^176^ While it is essential for maintaining retinal immune privilege at baseline, excessive activation can lead to retinal damage and inflammation.^177^ In AMD, increased levels of complement proteins such as C3a, C3b, and C5a are observed, which can intensify immune responses and contribute to the disease's progression.^178^, ^179^

Elevated complement C5a levels significantly stimulate T cells to produce IL‐22 and IL‐17, promoting the onset of AMD‐related inflammation.^180^ Considering the pivotal role of the complement system in the pathogenesis of AMD, novel adjunct therapies for both non‐neovascular and neovascular AMD could target complement factors and modulators. Further clinical trials are warranted to assess the durability of these treatments and to uncover any latent ocular adverse effects.^181^

### 4.3 Treatment

In our review, clear evidence supports the role of a range of pro‐inflammatory factors that are abundant in the ageing retina as driving forces in AMD pathogenesis. Insights from immunology are anticipated to play a significant part in future clinical management of AMD. The exclusive focus on angiogenesis inhibition with anti‐VEGF agents in conventional treatment may not be sufficient due to the inflammatory component in AMD pathology. Therapies incorporating anti‐inflammatory agents, such as corticosteroids, could be beneficial.^182^, ^183^, ^184^ Non‐steroidal anti‐inflammatory drugs (NSAIDs),^185^ immunosuppressive agents (e.g., methotrexate and rapamycin),^186^ and biologics (e.g., infliximab, daclizumab, and complement inhibitors)^187^ may provide an adjunct or alternative mechanism to suppress the inflammatory processes driving AMD progression. There are several clinical trials have been conducted recently to explore the effect of immunological treatment for AMD (Table 2). More studies are required to test the efficacy and safety of systemic, periocular, and intravitreal applications of each anti‐inflammatory agent.

**TABLE 2:** Clinical trials for age‐related macular degeneration (AMD) immune therapy in 2 years.

Study ID	Patients	Drugs	Phases	Year	Country
NCT00466076	Macular degeneration	Copaxone	III	2024	Israel
NCT00935883	AMD	Eculizumab	III	2024	USA
NCT00766649	AMD	Sirolimus	I	2024	USA
NCT00464347	AMD	Avastin, TAC‐PF	II	2024	USA
NCT00447031	Macular degeneration	Intravitreal bevacizumab TAC‐PF	NA	2024	Korea
NCT02357342	AMD	Sirolimus, anti‐VEGF	II	2024	USA
NCT00100009	Macular degeneration	TAC‐PF	III	2004	USA
NCT01249937	Wet macular degeneration	Ranibizumab, TAC‐PF	II	2024	Canada
NCT01445548	AMD	Sirolimus	I	2024	USA
NCT00857259	AMD	Everolimus, ranibizumab	II	2024	USA
NCT00370370	Neovascular AMD	Bevacizumab, TAC‐PF	III	2024	Iran
NCT00370539	Neovascular AMD	Verteporfin, bevacizumab, TAC‐PF	III	2024	Iran
NCT00712491	AMD, choroidal neovascularization	Sirolimus	II	2024	USA
NCT00304954	AMD, choroidal neovascularization	Daclizumab, infliximab, rapamycin	II	2024	USA
NCT00766337	AMD, choroidal neovascularization	Sirolimus, ranibizumab	II	2024	USA
NCT00242580	Macular degeneration, choroidal neovascularization	Pegaptanib, TAC‐PF	III	2024	USA
NCT00071227	Macular degeneration, retinal vein occlusion	TAC‐PF	I	2023	USA
NCT02806752	Wet macular degeneration	TAC‐PF, ranibizumab	IV	2024	China

Abbreviations: TAC‐PF, triamcinolone acetonide; VEGF, vascular endothelial growth factor.

*Source*: www.clinicaltrials.gov.

## 5 GRAVES’ OPHTHALMOPATHY

### 5.1 Definition and classification

GO, also known as thyroid eye disease or thyroid‐associated orbitopathy, is a rare and complex autoimmune disorder characterized by a high incidence. It can lead to disfigurement of the orbit, diplopia, and even vision loss. The most common clinical features of GO include upper eyelid edema, conjunctival and periorbital erythema, and proptosis. The majority (>90%) of patients with GO have Graves’ disease, an inflammatory autoimmune condition that is caused by thyrotropin (TSH) receptor (TSHR) autoantibodies (TSHRAbs).^188^ Graves’ disease is quite prevalent worldwide. This condition primarily affects women, typically between the ages of 30 and 50 years, with an overall prevalence of 0.5%. Among patients with Graves’ disease who do not present with GO, approximately 15% may develop GO within an average timeframe of 3‒6 months.^189^, ^190^

Currently, there are several classification systems for GO; however, the European Group on Graves’ Orbitopathy (EUGOGO) classification is widely accepted. The EUGOGO classification categorizes the severity of the disease into vision‐threatening, mild, moderate, and severe GO.^191^

### 5.2 Cellular mechanisms of immune response in Graves’ ophthalmopathy

#### 5.2.1 Orbital fibroblasts

Most of the signs and symptoms of GO can be attributed to the expansion of orbital contents. Orbital fibroblasts are targeted by a series of autoimmune responses, which collectively induce proliferation, excessive adipogenesis (leading to the differentiation of fibroblasts into new adipocytes), and overproduction of extracellular matrix, playing a dominant role in the pathogenesis of GO. Fibroblasts originating from the bone marrow differentiate into CD34^+^ fibroblasts, which can further differentiate into adipocytes or myofibroblasts. These CD34^+^ fibroblasts coexist with resident CD34^‒^ fibroblasts within the orbital tissue.^192^ Fibroblasts responded vigorously to various inflammatory mediators, including IL‐1β, IL‐6, IL‐17, TNF‐α, TGF‐β, and IFN‐γ. In GO patients, orbital fibroblasts demonstrated high expression of the costimulatory molecule CD40 when stimulated by IFN‐γ. Activation of human orbital fibroblasts through CD40 significantly induced the synthesis of hyaluronic acid (HA), exacerbating the swelling of orbital tissue. Additionally, the CD40‒CD154 interaction contributed to the physical interactions between orbital fibroblasts and T lymphocytes in GO, enhancing the expression of intercellular adhesion molecule‐1 and the production of cytokines and prostaglandins by orbital fibroblasts.^193^, ^194^, ^195^ IL‐17 can enhance the expression of RANTES in orbital fibroblasts through the CD40‒CD40L interaction, thereby promoting the pro‐inflammatory and pro‐fibrotic functions of orbital fibroblasts and exacerbating fibrosis in patients with GO.^196^, ^197^ These findings indicated that fibroblasts play an active role in regulating the inflammatory process of GO through the action of inflammatory factors.

#### 5.2.2 Immune cells

Immune cells such as T cells, B cells, and antigen‐presenting cells (APCs) play a crucial role in the pathogenesis of GO. These cells can release inflammatory cytokines including IL‐1, IL‐4, TNF‐α, and IFN‐γ.^198^ These cytokines facilitate a cascade of inflammatory responses, contributing to the characteristic symptoms and signs of GO, such as orbital tissue swelling, muscle inflammation, and alterations in ocular function. The interplay between these immune cells and the signaling pathways they activate underscored the complexity of the autoimmune processes underlying GO. An increasing body of evidence suggests that Th1 (cytotoxic) and Th2 (antibody‐producing) cell subsets, along with Th17 (fibrotic) cells, are all implicated in the pathogenesis of GO.^15^, ^199^ Fang et al.^200^, ^201^ found that pathogenic Th17 cells play a regulatory role in orbital fibrosis and adipogenesis in patients with GO, and the interaction between CCR6^+^ Th17 cells and CD34^+^ fibroblasts promote inflammation, exacerbating the condition of GO. The Th1/Th17 “mixed” phenotype is also associated with dysregulation of lipid metabolism in patients with severe GO.^202^ IFN‐γ has been extensively studied for its roles in GO. Secreted by Th1 cells, IFN‐γ could induce the activation of fibroblasts and upregulate CD40 expression on these cells, thereby influencing immune interactions. Additionally, IFN‐γ promoted the expression of hyaluronic acid synthase 2 (HAS2), enhancing the synthesis of HA in fibroblasts in response to IL‐1β. The accumulation of HA was closely associated with orbital tissue swelling observed in GO.^203^ Th2 cells secreted IL‐4, which also modulated the expression of HAS2 and inhibited the secretion of prostaglandin E2 (PGE2) from the orbital fibroblasts in cases of GO. This further promoted the synthesis of HA in fibroblasts when induced by IL‐1β, contributing to the progression of GO. Moreover, research by Kim et al.^203^, ^204^ has shown elevated levels of serum IL‐17A, IL‐23, and IL‐6 in patients with GO, highlighting the significance of the Th17 pathway and the IL‐23/IL‐17 axis in the progression of this disease. The interplay between these cytokines and the different T‐cell subsets underscored the complexity of the inflammatory responses involved in the pathogenesis of GO.

The role of B cells in GO should not be overlooked. Activated B cells differentiate into plasma cells that secrete TRAb. TRAb can be classified into several types: thyroid‐stimulating antibody (TSAb), thyroid‐blocking antibody (TBAb), and neutral antibodies.^205^ TSAb is associated with hyperthyroidism, as it induces downstream effects similar to the binding of TSH to its receptor, leading to thyroid cell proliferation, thyroid growth, and the secretion of thyroid hormones (T4 and T3), ultimately resulting in hyperthyroidism.^206^ In patients with GO, TSAb served as a specific biomarker and was implicated in various clinical manifestations. Furthermore, TSAb levels were closely related to the clinical activity and severity of GO.^207^ TRAb could also bind to insulin‐like growth factor 1 (IGF‐1) and TSHR present on the surface of thyroid epithelial cells and fibroblasts. This binding led to cellular activation and the release of inflammatory cytokines.^208^, ^209^

**FIGURE 3:**
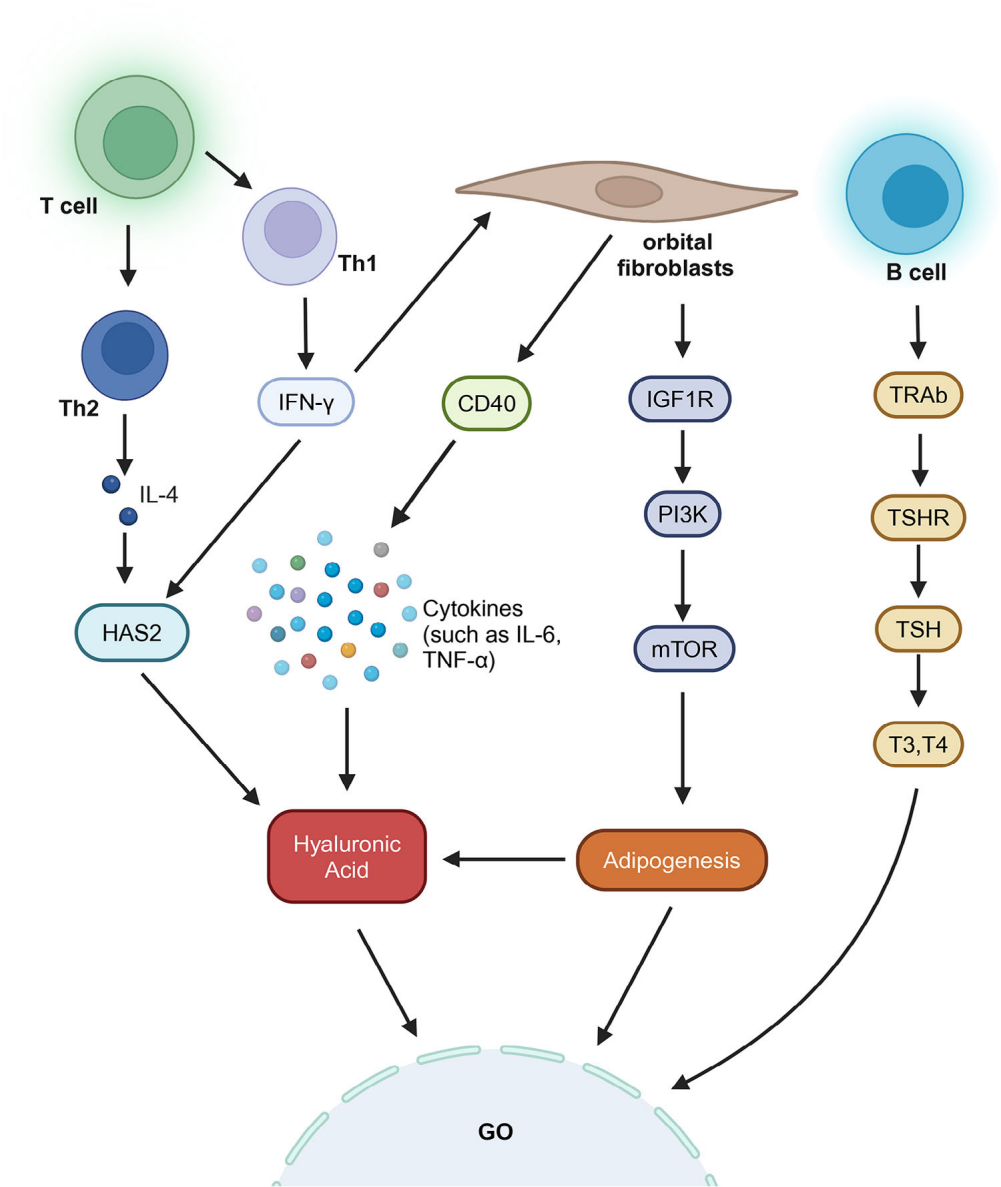
The pathogenic mechanisms of Graves’ ophthalmopathy (GO) regulated by multiple cell types. Orbital fibroblasts release cytokines through cluster of differentiation 40 (CD40), T helper 1 (Th1) and Th2 cells increase hyaluronan synthase 2 (HAS2) expression, and the insulin‐like growth factor 1 receptor (IGF1R)/phosphoinositide 3‐kinase (PI3K)/mammalian target of rapamycin (mTOR) pathway jointly promote the elevated synthesis of HA, contributing to the pathogenesis of GO. Activated B cells secrete thyroid stimulating hormone receptor antibodies (TRAb), which stimulate the secretion of thyroid hormones (T4 and T3), directly exacerbating the progression of GO. TSH, thyroid stimulating hormone; TSHR, thyroid stimulating hormone receptor.

TSHR and IGF1R also play crucial roles in the pathogenesis of GO. Research has shown that the disruption of TSHR self‐tolerance led to the recognition of TSHR epitopes by APCs and B cells, subsequently activating naïve Th cells. This finding highlighted the intricate connections between TSHR and various immune cells.^210^ Krieger et al.^211^ discovered that the concurrent activation of TSH and IGF‐1 synergistically enhanced the secretion of HA, further intensifying the progress of GO. Furthermore, nuclear Forkhead transcriptional factors (FOXOs) served as convergence points for TSHR and IGF1R signaling pathways in GO. As inhibitory factors, FOXOs protected orbital fibroblasts from excessive adipogenesis and the overproduction of HA through the IGF1R‒PI3K‒Akt and mTORC1 signaling pathways.^211^ These combined factors contributed to the development of GO (Figure 3).

### 5.3 Treatment

There are various approaches to treating GO, but corticosteroids remain the most primary and widely used therapeutic agents. The European Thyroid Association (ETA) recommended IV corticosteroid pulse therapy as the first‐line treatment for active, moderate to severe thyroid‐associated ophthalmopathy. They suggested a cumulative dosage of 4.5 g of IV methylprednisolone administered over 12 weeks.^212^, ^213^ For example, methylprednisolone could inhibit the secretion of prostaglandins, fibroblast activity, and HA production, as well as the expression of pro‐inflammatory proteins in orbital tissues. Additionally, high‐dose methylprednisolone reduced the number of circulating dendritic cells and lowered levels of TSHRAb, which also played a crucial role in the management of Graves’ disease.^214^, ^215^ Although corticosteroids showed significant efficacy in the treatment of GO, the associated side effects could not be overlooked, creating an urgent need for alternative therapeutic strategies. Currently, for patients who were insensitive or unresponsive to corticosteroid treatment, surgical intervention—specifically orbital decompression surgery—and ocular radiation therapy represented viable options.^216^, ^217^

However, as research into the underlying mechanisms progresses, the advantages of certain targeted therapies have become increasingly apparent. Etanercept, a recombinant DNA‐derived drug, bound to TNF‐α, inhibiting its role in various autoimmune diseases. Studies have shown that etanercept demonstrates notable alleviation and therapeutic effects in patients with GO.^218^ The Food and Drug Administration has approved adalimumab for the treatment of numerous autoimmune diseases, and it has also shown significant efficacy in the management of GO.^219^ Tocilizumab, a humanized monoclonal antibody targeting IL‐6R, was utilized in several autoimmune conditions. Research involving patients with GO has revealed that tocilizumab was effective and significantly aids in the treatment of refractory GO, as well as in preventing relapse.^219^ Turcu et al.^220^ identified a small TSH receptor antagonist (NCGC00229600) that resulted in reduced HA production in retro‐orbital fibroblasts/adipocytes, showing potential therapeutic effects for GO. Furthermore, Chen et al.^221^ discovered teprotumumab (RV 001 and R1507), a human monoclonal antibody, that blocks IGF1R. This agent attenuates the effects of IGF‐1 and TSH in orbital fibroblasts and inhibits the expression of pro‐inflammatory cytokines IL‐6 and IL‐8 induced by TSH. Belimumab was a monoclonal antibody targeting B‐cell activating factor (BAFF) that directly interacted with B cells and indirectly reduced B‐cell survival by blocking the interaction between BAFF and its receptor, thereby decreasing the production of TRAb and alleviating GO.^222^, ^223^

In addition to these targeted therapies, antithyroid drugs have been widely recommended as a fundamental therapeutic approach for patients worldwide. Their mechanism of action involved the inhibition of iodination, which subsequently reduced the production of thyroid hormones, thereby suppressing the progression of GO.^219^

Although a variety of pharmacological treatments are available for GO, the primary concern remains the associated side effects. Therefore, the investigation of new therapeutic options that are both effective and have a lower risk of adverse effects continues to be a crucial focus of research at present.

## 6 CONCLUSION AND PROSPECTS

Since, extensive research has been conducted on immune‐related ocular diseases; however, many questions remain unresolved. First, one of the primary issues is the complexity of the pathogenic mechanisms involved in these diseases. Under any specific condition, it is still not entirely clear which mechanism plays a dominant role. Second, in terms of clinical management, while drugs developed to target immune mediators have shown some efficacy, for certain patients, the therapeutic potency of those drugs is still suboptimal. These patients often require higher doses of corticosteroids, which poses significant concerns due to the side effects associated with long‐term use. Therefore, there is an urgent need to develop new therapeutic approaches to safeguard the health of these patients.

Ocular immune‐related diseases, including but not limit to uveitis, AMD, DR, and GO, are characterized by a complicated interaction among different types of immune responses. The role of immune cells and their derived cytokines has been central to the pathogenesis of these conditions. The associated treatment strategies are also developed from these pathophysiological mechanisms.

The treatment strategies to uveitis have shifted from traditional corticosteroids and immunosuppressants to precise medicine targeting specific molecular pathways. For instance, therapies targeting the Janus kinase (JAK)/STAT pathway have shown potential in treating uveitis. DR treatment strategies include laser photocoagulation, anti‐VEGF biologics, and glycemic control. Future research directions may focus on improving the treatment of diabetes itself to reduce the development of DR. AMD treatment strategies include anti‐VEGF management, photodynamic therapy, and nutritional supplementation. With a deeper understanding of the molecular mechanisms of AMD, future treatments may become more personalized, targeting specific genetic and environmental risk factors. GO treatment strategies include immunosuppressants, corticosteroids, and therapies targeting the TGF‐β signaling pathway. Future research may explore more biomarkers and therapeutic targets to improve the effectiveness of treatments for GO. In terms of molecular mechanisms, all four types of diseases involve inflammatory responses, but the specific molecular pathways and mediators are different. In terms of therapeutic strategies, while immunosuppressants may be used for all, targeted therapies for specific molecular pathways, such as anti‐VEGF therapy and targeted TGF‐β therapy, show the potential for more precise treatments for specific diseases. Thus, immunotherapy, particularly targeted those regulating checkpoints, offers new directions for managing these challenging diseases.

Looking into the future, the application of immunotherapy in ocular immune diseases holds broad prospects. With an increasing understanding of the immunopathology of certain diseases, a personalized as well as precision medicine may become possible. For instance, T‐cell‐based therapies improved by gene‐editing technologies have shown potential not only in oncology but also in autoimmune diseases. Precision and personalization in immunotherapy will enable treatment regimens to be optimized for individual patient profiles, enhancing therapeutic efficacy and reducing adverse reactions. However, challenges remain in regarding to improve efficacy, to minimize side effects, and to incorporate into the existing systems. Future studies need to further delineate the mechanisms of these therapies, to optimize timing, dosing and route of administration, and to monitor potential changes following treatment. As technology advances and clinical experience accumulates, there is reason to believe that immunotherapy will bring renewed hope for patients with ocular diseases (Table 3).

**TABLE 3:** Comparison of molecular mechanisms and therapeutic strategies.

Disease	Molecular mechanisms	Therapeutic strategies
Uveitis	Involves various inflammatory mediators and autoimmune responses	Targeted JAK/STAT pathway, immunosuppressants, biologics
DR	Vascular damage, inflammation, and neovascularization	Laser photocoagulation, anti‐VEGF drugs, glycemic control
AMD	Degenerative changes in the macula, involving inflammation and oxidative stress	Anti‐VEGF therapy, photodynamic therapy, nutritional supplementation
GO	Orbital tissue remodeling and fibrosis, involving TGF‐β signaling pathway	Immunosuppressants, corticosteroids, targeted TGF‐β therapy

Abbreviations: AMD, age‐related macular degeneration; DR, diabetic retinopathy; GO, Graves’ ophthalmopathy; JAK, Janus kinase; STAT, signal transducer and activator of transcription; TGF‐β, transforming growth factor‐beta; VEGF, vascular endothelial growth factor.

## AUTHOR CONTRIBUTIONS

Shengping Hou reviewed and edited this manuscript. Yakun Wang and Shangze Gao wrote the original draft. Fan Cao, Hui Yang, and Fengyang Lei collected literature and provided figures. All authors have read and approved the article.

## CONFLICT OF INTEREST STATEMENT

The authors declare they have no conflicts of interest.

## ETHICS STATEMENT

Not applicable.

## Data Availability

Not applicable.
